# Conditional Knockout of N-WASP Enhanced the Formation of Keratinizing Squamous Cell Carcinoma Induced by KRas^G12D^

**DOI:** 10.3390/cancers15184455

**Published:** 2023-09-07

**Authors:** Pazhanichamy Kalailingam, Apoorva Verma, Ying Hui Lee, Thirumaran Thanabalu

**Affiliations:** School of Biological Sciences, Nanyang Technological University, 60 Nanyang Drive, Singapore 637551, Singapore; pazhanichamy.k@ntu.edu.sg (P.K.); apoorva1712@gmail.com (A.V.); yinghui001@e.ntu.edu.sg (Y.H.L.)

**Keywords:** N-WASP, keratin pearl, skin cancer

## Abstract

**Simple Summary:**

The expression of N-WASP (Neural Wiskott-Aldrich Syndrome Protein) is found to be reduced in certain human cancers, and this has been associated with poor prognoses for patients. To characterize the role of N-WASP in the tumorigenesis of squamous cell carcinoma (SCC), we generated mice which expressed a mutant oncogene with (N-WASP^HetG12D^) or without (N-WASP^KOG12D^) the expression of N-WASP upon Tamoxifen (TAM) injection. Both sets of mice had similar weights and did not display any abnormalities prior to the TAM injections. The N-WASP^KOG12D^ mice displayed significantly greater weight loss and hair loss along the dorsal skin post-injection when compared to the N-WASP^HetG12D^ mice. The N-WASP^KOG12D^ mice developed tumors 2 weeks after their exposure to TAM, with 73% of these mice succumbing to the disease within 6 weeks of the injection. Subsequently, the remaining mice in this group died within 7 to 8 weeks post injection. On the contrary, the N-WASP^HetG12D^ mice developed tumors only after 7 weeks following the injection and survived for up to 116 days. Our findings reveal that N-WASP deficiency activates signaling pathways which enhance cell proliferation. An N-WASP deficiency led to the acceleration of mutant-oncogene-induced SCC formation in mice, suggesting a tumor suppressor role for N-WASP in skin cancer. Thus, N-WASP could potentially serve as a novel marker for the development of skin cancer and the pathogenesis of SCC.

**Abstract:**

Squamous cell carcinoma (SCC) is one of the most common forms of skin cancer in humans, and Neural Wiskott-Aldrich Syndrome Protein (N-WASP) plays a crucial role in epidermal homeostasis. To elucidate the role of N-WASP in skin cancer, we generated mice which expressed constitutively active KRas (KRas^G12D^) in keratinocytes with either homozygous (N-WASP^KOG12D^) or heterozygous (N-WASP^HetG12D^) N-WASP knockout upon Tamoxifen (TAM) injection. Both the N-WASP^KOG12D^ and N-WASP^HetG12D^ mice had similar body weights and no congenital malformations prior to the injection of TAM. Within 2 weeks of the injections, the N-WASP^KOG12D^ mice exhibited significant reductions in weight coupled with visible tumors at numerous sites, unlike the N-WASP^HetG12D^ mice, which had no visible tumors. We found that both sets of mice had oily, sticky skin and wet eyes 3 weeks after their exposure to TAM, indicating the overproduction of sebum/meibum. At 37 days post TAM injection, several notable observations were made. Tumors collected from the N-WASP^KOG12D^ mice had small- to large-sized keratin pearls that were not observed in the N-WASP^HetG12D^ mice. A Western blot and immunostaining analysis both highlighted significantly higher levels of expression of SCC markers, such as the cytokeratins 8, 17, 18, and 19 and TP63, in the tumors of the N-WASP^KOG12D^ mice compared to those of the latter group. Furthermore, we noted increases in the expression levels of EGFR, P-ERK, GLUT1, P-mTOR, and P-4EBP in the N-WASP^KOG12D^ mice, suggesting that the deletion of N-WASP in the keratinocytes enhanced KRas signaling and glucose uptake, resulting in aggressive tumor formation. Interestingly, a thickening of the epidermal layer within the esophagus and tongue was only observed in the N-WASP^KOG12D^ mice. Immunostaining for PCNA emphasized a significantly higher number of PCNA-positive cells in the skin of the N-WASP^KOG12D^ mice compared to their counterparts, implying that epidermal thickening and enhanced tumorigenesis are due to an increased proliferation of keratinocytes. Through our results, we have established that N-WASP plays a tumor-suppressive role in skin cancer.

## 1. Introduction

Most cancers arise from the epithelial cells of the skin, esophagus, lung, colon, breast, and prostate and account for millions of deaths per year [1,2]. Squamous cell carcinoma (SCC) is one of the most common forms of skin cancer, occurring predominantly in sun-exposed regions of the skin [3]. Annually, more than 1 million cases of SCC are diagnosed in the US alone. To date, early detection and cutaneous surgical excision remain the most effective methods of treatment for SCC. However, about 8% of treated patients experience a cancer relapse and in about 5% of these cases, the SCC metastasizes to distant sites within 5 years [4]. In patients with metastatic SCC, the prognosis is very poor, with only a 10–20% survival rate over 10 years [4,5]. Genetic alterations in the RAS and P53 genes are commonly identified in aggressive SCC [6]. Mutations in the RAS genes are found in approximately 10–30% of human skin SCC [7], while KRas^G12D^ mutations have also been widely identified in skin SCC [5]. 

N-WASP is a ubiquitously expressed protein which plays a vital role in actin cytoskeleton remodeling by activating the Arp2/3 complex, a known actin nucleator [8]. N-WASP has been shown to be a key regulator in the maintenance of skin homeostasis [9,10]. The knockout of N-WASP in mouse skin fibroblasts (using FSP1-Cre) resulted in enhanced wound healing and the activation of the TGF-β [10] pathway, while knocking out N-WASP in mouse keratinocytes (using K14-Cre) led to a skin barrier defect and the hyperproliferation of the keratinocytes [9]. The ablation of N-WASP in keratinocytes activates Wnt signaling, which regulates hair follicle cycling via the TGF-β pathway [11]. Our group previously demonstrated that N-WASP plays a pivotal role in cell adhesion, cell spreading [12], and signaling in epithelial tissues [9,10]. Moreover, N-WASP is essential for the maintenance of the blood–testis barrier [13] and vascular permeability in the lungs [14]. WASP family proteins are important factors in regulating actin polymerization during cell migration and invasion [15]. Earlier studies found increased levels of expression of N-WASP in pancreatic carcinoma, liver adenocarcinoma [16,17], and invasive ductal breast carcinoma [18]. N-WASP has also been noted to be highly expressed in Stage 3 and Stage 4 lung cancer biopsies, correlating with reduced patient survival [19]. Another study has shown that N-WASP is highly expressed in cervical cancer and stimulates the invasion and migration of cervical cells via P38 MAPKs signaling [20]. Conversely, N-WASP expression has been reported to be reduced in clear cell renal carcinoma [21], while Martin et al., 2011 also put forward that the decreased expression of N-WASP in colorectal cancer was associated with poor patient prognosis [22]. A meta-analysis of published microarray datasets of lung cancer showed that reduced N-WASP expression was associated with a reduced survival rate in Stage 1 lung cancer patients (n = 577). A recent study posited that the loss of N-WASP in an Apc-deletion mouse model promotes intestinal tumorigenesis and a decreased rate of survival, proposing that N-WASP is a tumor suppressor in colorectal cancer [23]. However, the role of N-WASP in epithelial skin cancer remains unclear.

In this report, we determined the role of N-WASP in epidermal SCC using N-WASP^KOG12D^ and N-WASP^HetG12D^ mice in which the conditional activation of KRas^G12D^ expression and ablation of the N-WASP (homozygous or heterozygous knockout) in keratinocytes were achieved via TAM injection. Within 2 weeks of the injections, the N-WASP^KOG12D^ mice were found to have significant reductions in weight and increased hair loss along the dorsal skin when compared to the N-WASP^HetG12D^ mice. Tumors appeared first on the lips, face, anus, palm/soles, eyelids, and ears of the N-WASP^KOG12D^ mice within just 2 weeks of the injection, while tumors in the same sites appeared on N-WASP^HetG12D^ mice only 37 days after the injection, implying that the homozygous knockout of N-WASP accelerated the KRas^G12D^-induced formation of SCC in the mice as opposed to the heterozygous knockout. Additionally, we recorded a higher number of keratin pearls (ranging from small to large in size) and enhanced levels of expression of SCC markers (such as K8, K17, K18, TP63, and K19) in the N-WASP^KOG12D^ mice in contrast with the N-WASP^HetG12D^ group. These results indicate that N-WASP probably acts as a tumor suppressor in skin cancer and could be a novel marker for the development of skin cancer.

## 2. Materials and Methods

### 2.1. Animals

To generate the N-WASP^fl/fl^; KRas^G12D^ mice, we crossed N-WASP^fl/fl^ (female) mice with KRas^G12D^ (male) mice to obtain N-WASP^WT/fl^; KRas^G12D^ mice. These mice were then crossed with N-WASP^fl/fl^ mice to obtain N-WASP^fl/fl^; KRas^G12D^ mice. Subsequently, keratinocyte-specific N-WASP knockout mice were generated by crossing N-WASP^fl/fl^; KRas^G12D^ (female) mice with N-WASP^WT/fl^; K5-CreERT (male) mice. The heterozygous N-WSAP^WT/fl;^ KRas^G12D^; K5-CreERT (N-WASP^HetG12D^) and homozygous N-WASP^fl/fl^; KRas^G12D^; K5-CreERT (N-WASP^KOG12D^) mice obtained from the above crosses were used for further experiments. A PCR was performed to detect flox/flox and Cre within the genomic DNA of the tails of the mice, with primers as described in Kalailingam et al. [9]. The primers used to detect KRas^G12D^ were GTCTTTCCCCAGCACAGTGC, CTCTTGCCTACGCCACCAGCTC, and AGCTAGCCACCATGGCTTGAGTAAGTCTGCA. The mice were maintained on a standard chow diet with unlimited water at a constant temperature of 23°C, and with a 12 h/12 h artificial dark/light cycle. All animal experiments were carried out with the approval of the Institutional Animal Care and Use Committee (IACUC) of Nanyang Technological University (NTU) (ARF-SBS/NIE-A0386). 

### 2.2. Tamoxifen Injection

Tamoxifen (TAM) was dissolved in corn oil at a concentration of 12 mg/mL via shaking overnight at 37 °C. Approximately 75 mg of TAM/kg body weight was injected into 5-week-old mice over the course of three days. N-WASP deletion was then confirmed by conducting a PCR using tail genomic DNA from the mice, using another reverse primer (WASL-Flr) paired with the flox/flox 5′ primer GGACAGGGTCTATCTCTGAATTCCT. 

### 2.3. Hematoxylin and Eosin (H&E) and Toluidine Blue Staining and Immunostaining

The N-WASP^HetG12D^ and N-WASP^KOG12D^ mice were anesthetized and sacrificed via CO_2_ euthanization. Skin samples from the dorsal regions, ears and tails of the mice were processed as previously described [9]. The images were acquired using an Olympus microscope(Olympus Corporation of the Americas, Coopersburg, PA, USA) with a CoolSNAPHQ2 camera (TELEDYNE PHOTOMETRICS 3440 E. Britannia Drive, Suite 100, Tucson, AZ 85706, USA). The dilutions of the antibodies used were carried out as follows: Involucrin, K5, K10, PCNA, CD11b, SMA, CD3, and CD4 (1:100, Santa Cruz, Dallas, TX, USA.), K17, K8, K19, and K17 (1:50, DSHB Iowa, USA), Ly6G/Ly6C (1:100, Biolegend, San Diego, CA, USA), E-cadherin (1:100, BD biosciences, Singapore), K14 (1:10, LL001, a from gift from Birgit’s lab), and Alexa Fluor 594 Goat Anti-rabbit (1:50, Molecular Probes Life Tec Mass, Waltham, MA, USA).

### 2.4. Oil Red O Staining

At room temperature, the tissue section slides were washed with PBS, fixed in a 10% formalin solution for 10 min, and then incubated for 30 min with a 60% filtered Oil Red O stock solution (0.3 g/100 mL of isopropanol). The slides were thoroughly washed with 60% (vol/vol) isopropanol and water before visualization. The images were captured using an Olympus microscope with 20× and 40× objective lenses. The intensity of the Oil Red O staining was qualified using ImageJ software (Version 1.8.0). 

### 2.5. Statistical Analysis

A statistical analysis was performed using data from three independent experiments via Student’s t-test, and *p* < 0.05 was considered statistically significant. Values presented in bar charts represent the mean ± SD of each value. 

## 3. Results

### 3.1. The Homozygous Deletion of N-WASP Increased the Tumor Burden and Decreased the Survival of Mice with KRas^G12D^-Induced Tumor

We and Luminov et al., 2010 previously established that the ablation of N-WASP in keratinocytes causes epidermal hyperplasia in mice due to the increased proliferation of keratinocytes [9,24], and the expression of N-WASP has been found to be reduced in several cancers [21,22,25]. To illuminate the role of N-WASP in skin cancer, we generated mice with the TAM-inducible, keratinocyte-specific homozygous or heterozygous N-WASP deletion and expression of KRas^G12D^. In brief, we crossed N-WASP^fl/fl^ mice with LSL-KRas^G12D^ (LSL: Loxp-Stop-Loxp) mice to obtain N-WASP^WT/fl^; KRas^G12D^ mice (Figure S1A; LSL-KRas^G12D^: 500 bp, WT KRas: 700 bp). The N-WASP^WT/fl^; KRas^G12D^ mice were crossed with N-WASP^fl/fl^ mice to generate N-WASP^fl/fl^; KRas^G12D^ mice (Figure S1B). The N-WASP^fl/fl^; KRas^G12D^ mice were finally crossed with N-WASP^WT/fl^; K5-CreERT mice to generate N-WASP^fl/fl^; KRas^G12D^; K5-CreERT (N-WASP^KOG12D^) mice and N-WASP^WT/fl^; KRas^G12D^; K5-CreERT (N-WASP^HetG12D^) mice. Twenty-five percent of the pups born were N-WASP^KOG12D^, which is consistent with the expected Mendelian ratio (Figure S1C). The TAM-induced excisions of the exons 3 and 4 of the N-WASP gene were confirmed via a PCR using tail genomic DNA from the N-WASP^KOG12D^ and N-WASP^HetG12D^ mice (Figure S1D). We confirmed the deletion of N-WASP and the expression of the KRas mutant (KRas^G12D^) within the epidermis via a Western blot analysis, using epidermal protein extracted from the skin of the N-WASP^KOG12D^ and N-WASP^HetG12D^ mice (Figure S1E).

**Figure 1:**
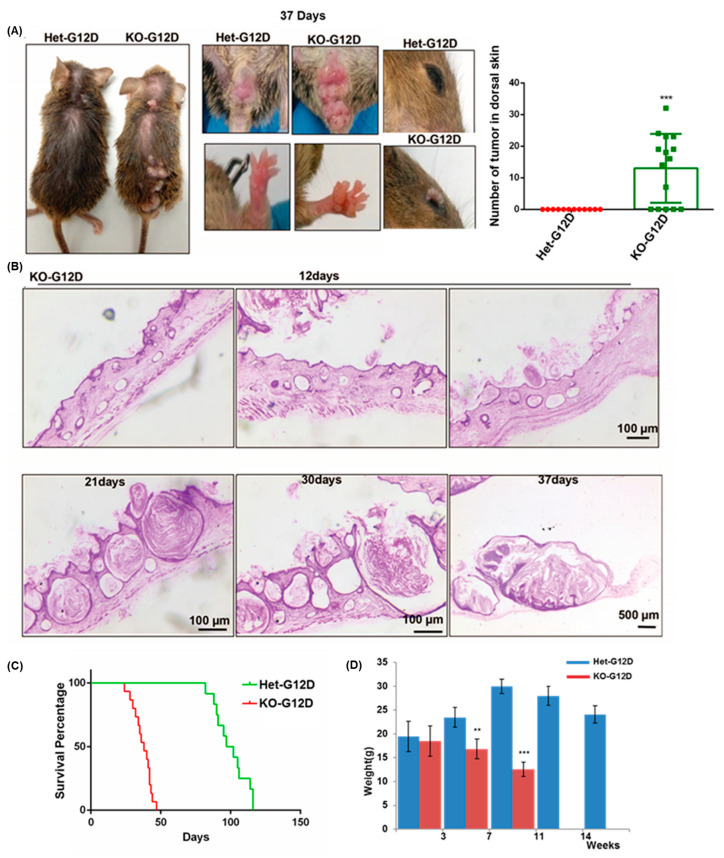
The ablation of N-WASP in mice expressing KRas^G12D^ enhanced tumorigenesis, increased the tumor burden, and decreased survival. (**A**) Tumors appeared on the dorsal skin, lips, face, anus, and palm/soles of the N-WASP^KOG12D^ and N-WASP^HetG12D^ mice at 37 days post TAM injection. The graph represents the number of tumors within the dorsal skin sections of the N-WASP^KOG12D^ and N-WASP^HetG12D^ mice; (**B**) H&E-stained dorsal skin sections of N-WASP^KOG12D^ mice at 12 (n = 5), 21 (n = 5), 30 (n = 5), and 37 (n = 11) days post TAM injection. (**C**) The graph represents the survival curve of the N-WASP^KOG12D^ (n = 15) and N-WASP^HetG12D^ (n = 12) mice following the TAM injection. (**D**) Average body weight of N-WASP^KOG12D^ and N-WASP^HetG12D^ mice after TAM injection (n = 5). Results: means ± SDs. *** *p* < 0.001; ** *p* < 0.01.

Preceding the injections, both groups of mice were similar in size and weight and exhibited no external signs of congenital malformations. Two weeks post TAM injection, 60% (n = 9/15) of the N-WASP^KOG12D^ mice displayed hair loss in the middle of the dorsal skin which was not noted in the N-WASP^HetG12D^ mice. Tumors appeared on the N-WASP^KOG12D^ mice as early as 2 weeks after the TAM injections compared to the latter group, in which tumors appeared only after 37 days. The initial tumors appeared in non-hair-bearing areas on the N-WASP^KOG12D^ mice, such as the lips (53% of the animals treated (n = 8/15)), face (86%, n = 13/15), anus (100%, n = 15/15), palm/soles (100%, 15/15), eyelids (53%, n = 8/15), and ears (13%, n = 2/15) before appearing on the dorsal skin (60%, 9/15) (Figure 1A, Table 1). On the other hand, the N-WASP^HetG12D^ mice developed tumors in similar sites only after 37 days, including on the lips (50%, n = 6/12), face (41%, n = 5/12), anus (100%, n = 12/12), palm/soles (41%, 5/12), eyelids (50%, n = 6/12), and ears (8%, n = 1/12). Notably, none were detected on the dorsal skin even after 116 days post injection. Hematoxylin and Eosin (H&E) staining revealed the formation of small-to-large keratin cysts and tumors on the dorsal skin of the N-WASP^KOG12D^ mice at 12, 21, 30, and 37 days after the TAM injection (Figure 1B). Small papillomas developed along the dorsal skin of the N-WASP^KOG12D^ mice within 12 days of exposure to TAM, appeared to have gradually increased by 21 days post-injection, and eventually resulted in SCC at the 37-day mark. Contrariwise, no papillomas were detected in the skin of the N-WASP^HetG12D^ mice, even up to 116 days after the TAM injection (Figure S2A–F). We found that the N-WASP^KOG12D^ mice developed skin tumors with an incidence of 100% and survived until 48 days post injection, while the N-WASP^HetG12D^ mice survived until 116 days post injection (Figure 1C and Figure S2A–F). We also observed a significant reduction in the weight of the N-WASP^KOG12D^ mice when compared to the N-WASP^HetG12D^ mice from 2 weeks after the injection of TAM (Figure 1D). Considering these results, we postulate that the homozygous deletion of N-WASP in the epidermis accelerates tumor formation while decreasing the rate of survival in mice with KRas^G12D^-induced tumors. 

### 3.2. The Deletion of N-WASP Expression and the Activation of KRas^G12D^ Expression Led to the Formation of Small or No Sebaceous Glands in the Skin of N-WASP^KOG12D^ Mice

The expression of the KRas^G12D^ mutant in various compartments of the skin, such as the epidermis, sebaceous glands (SGs), and hair follicular progeny induced tumor growth, SG abnormality, and keratin cyst formation [5]. We found that both the N-WASP^HetG12D^ and N-WASP^KOG12D^ mice developed oily, sticky skin and wet eyes 3 weeks after TAM injection, underscoring an overproduction of sebum/meibum. In stark contrast to the N-WASP^HetG12D^ mice, however, the oily skin phenotype was progressively reduced in the skin of the N-WASP^KOG12D^ mice, followed by an increase in the appearance of small papillomas.

**Figure 2:**
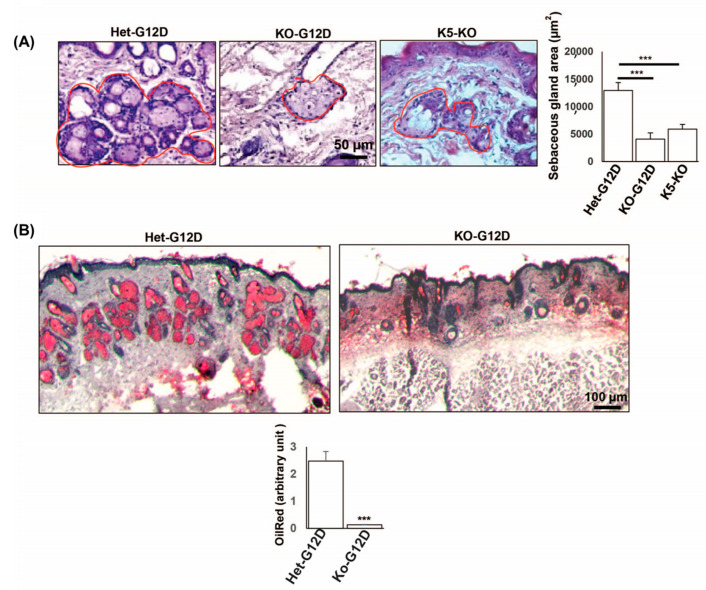
Ablation of N-WASP and expression of KRas^G12D^ caused defects in the formation of sebaceous glands. (**A**) H&E-stained dorsal skin sections of N-WASP^KOG12D^ and N-WASP^HetG12D^ mice at 37 days post TAM injection (n = 11) (Red circle is sebaceous gland). Quantification of sebaceous glands (SG) in N-WASP^KOG12D^, N-WASP^HetG12D^, and N-WASP^KO^ mice at 37 days post TAM injection (n = 5); (**B**) Oil-Red-O-stained dorsal skin sections of N-WASP^KOG12D^ and N-WASP^HetG12D^ mice at 37 days post TAM injection (n = 3). The graph represents the intensity of the Oil Red O staining of the dorsal skin sections of the N-WASP^KOG12D^ and N-WASP^HetG12D^ mice. Results: means ± SDs. *** *p* < 0.001.

Severely oily, sticky skin was observed throughout the lifespan of the N-WASP^HetG12D^ mice and could be correlated with changes in the SGs. To understand why this phenotype appeared reduced in the skin of the N-WASP^KOG12D^ mice when compared to their counterparts, we characterized the histological changes caused by the ablation of the expression of N-WASP in cells expressing KRas^G12D^ within the SGs. H&E staining was performed on the skin of the N-WASP^KOG12D^ and N-WASP^HetG12D^ mice at 12, 21 (Figure S3), and 37 days post TAM injection (Figure 2A). 

The H&E staining showed an abnormal pilosebaceous unit and dilated, hyperkeratinized infundibula in the dorsal skin of the N-WASP^KOG12D^ mice. These abnormal structural changes in the infundibula unit of the skin were due to altered epidermal terminal differentiation. The hyperkeratinized infundibula was associated with the formation of keratin cysts of varying sizes and the disappearance of or a reduction in the size of the SGs. However, we observed a normal infundibula (not keratinized) unit with an enlarged SG in the N-WASP^HetG12D^ mice (Figure 2A) at 37 days post TAM. 

These results indicate that abnormal epidermal terminal differentiation leads to changes in the entire infundibula unit, giving rise to either the absence of SGs or smaller SGs in the skin of the N-WASP^KOG12D^ mice. We also recorded decreased expression levels of transglutaminase and involucrin in N-WASP^KOG12D^ mice as opposed to the N-WASP^HetG12D^ mice (Figure S7), reiterating that abnormal terminal differentiation has the potential to cause prominent changes in the entire infundibula unit.

Our findings were consistent with the increased secretion of sebum in the mice, causing the oily, sticky skin phenotype. As previously mentioned, we noticed a gradual reduction of this phenotype in the N-WASP^KOG12D^ mice when compared to the N-WASP^HetG12D^ mice after 3 weeks post injection. Oil Red O staining further confirmed that the latter had a higher level of the production of lipids (Figure 2B). 

### 3.3. The Ablation of N-WASP Expression and the Activation of KRas^G12D^ Expression Led to the Formation of Keratin Cysts

**Figure 3:**
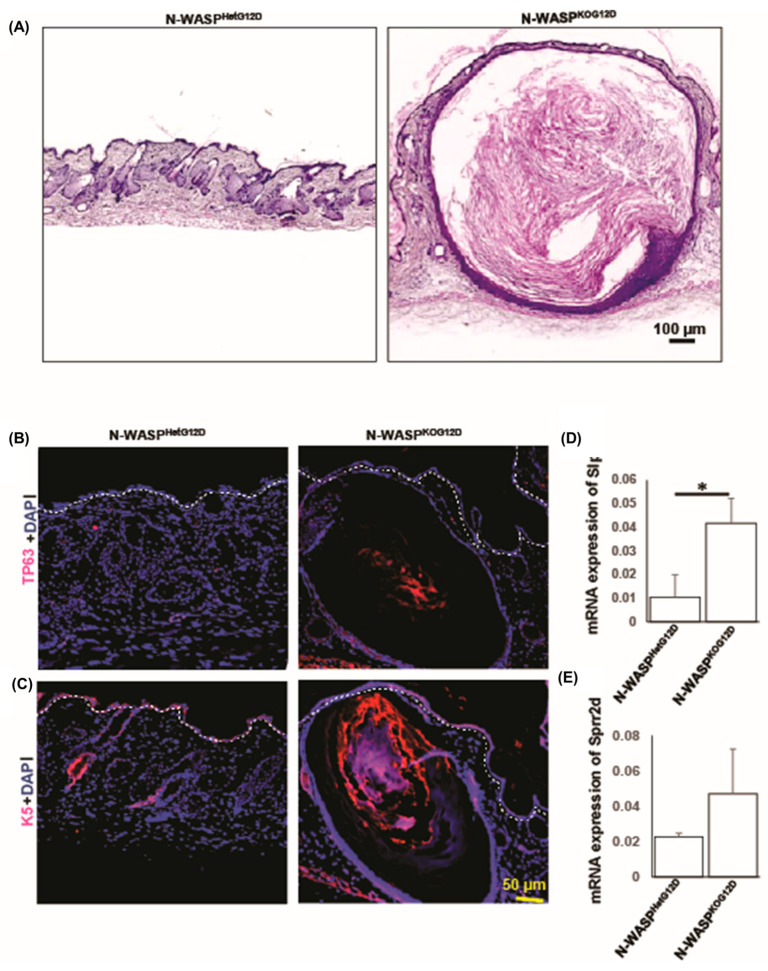
The ablation of N-WASP and the expression of KRas^G12D^ led to the formation of keratin cysts with high levels of expression of keratins. (**A**) H&E-stained dorsal skin sections of the N-WASP^KOG12D^ and N-WASP^HetG12D^ mice at 37 days post TAM injection (n = 11); (**B**,**C**) Immunostaining of TP63 and cytokeratin 5 in paraffin-embedded skin tissues of the N-WASP^KOG12D^ and N-WASP^HetG12D^ mice at 37 days post TAM injection (n = 3); (**D**,**E**) Using Trizol, the total RNA was isolated from the skin tumor samples of the N-WASP^KOG12D^ and N-WASP^HetG12D^ mice at 37 days post TAM injection, converted into cDNA, and used to performed a qPCR with Slpi and Sprr2d in the N-WASP^KOG12D^ and N-WASP^HetG12D^ mice after 7 weeks post TAM injection (n = 3). The expression level of GAPDH was used for normalization. Results: means ± SDs. * *p* < 0.05.

The over-production of keratins in the epidermis led to the formation of a hyperkeratinized cyst in the dermal region of the skin. The H&E staining of the dorsal skin of the N-WASP^KOG12D^ mice showed features of acanthosis, hyperkeratosis, and dilated infundibula, though these were not observed in that of the N-WASP^HetG12D^ mice (Figure 2A). In addition, keratinized infundibular cysts of various sizes were microscopically observed in the dorsal skin of the N-WASP^KOG12D^ mice at days 12, 21, and 37 post TAM injection (Figure S3C and Figure 3A). H&E and oil red staining for the skin of the N-WASP^KOG12D^ mice revealed that the larger keratin cysts contained lesser amounts of lipids and significantly smaller numbers of SGs. Moreover, these enlarged keratin cysts were associated with the over-production and abnormal accumulation of epithelial keratins. 

Previous results demonstrated that a thickening of the epidermis, hyperkeratosis, and dilated infundibula are due to the upregulation of the expression of Slpi, Sprr2d, and Epgn (Epigen) [26]. Our RT-PCR results showed an increase in the expression levels of Slpi and Sprr2d (no changes observed in Epgn) in the N-WASP^KOG12D^ mice compared to the N-WASP^HetG12D^ mice at 37 days post TAM (Figure 3D,E). Past reports have proposed that keratin cysts consist of different keratins (K10 and K19), TP63, and cyclin B1 [27,28,29]. To characterize the keratin cysts, we performed immunostaining for K10 and TP63 (Figure 3B,C), as well as a Western blot (Figure S4). The Western blot revealed that the expression levels of K10, TP63, and cyclin B1 were higher in the skin of the N-WASP^KOG12D^ mice when compared to that of the N-WASP^HetG12D^ mice (Figure S4A,B). In conclusion, the abnormal expression of Slpi and Sprr2d causes an increase in the expression levels of keratins in the epidermis and infundibula, leading to the dilation of the infundibula and the subsequent formation of enlarged keratin cysts in the skin of the N-WASP^KOG12D^ mice.

### 3.4. The Homozygous N-WASP Deletion in KRas^G12D^-Expressing Mice Induced the Formation of Aggressive Squamous Cell Carcinoma (SCC)

**Figure 4:**
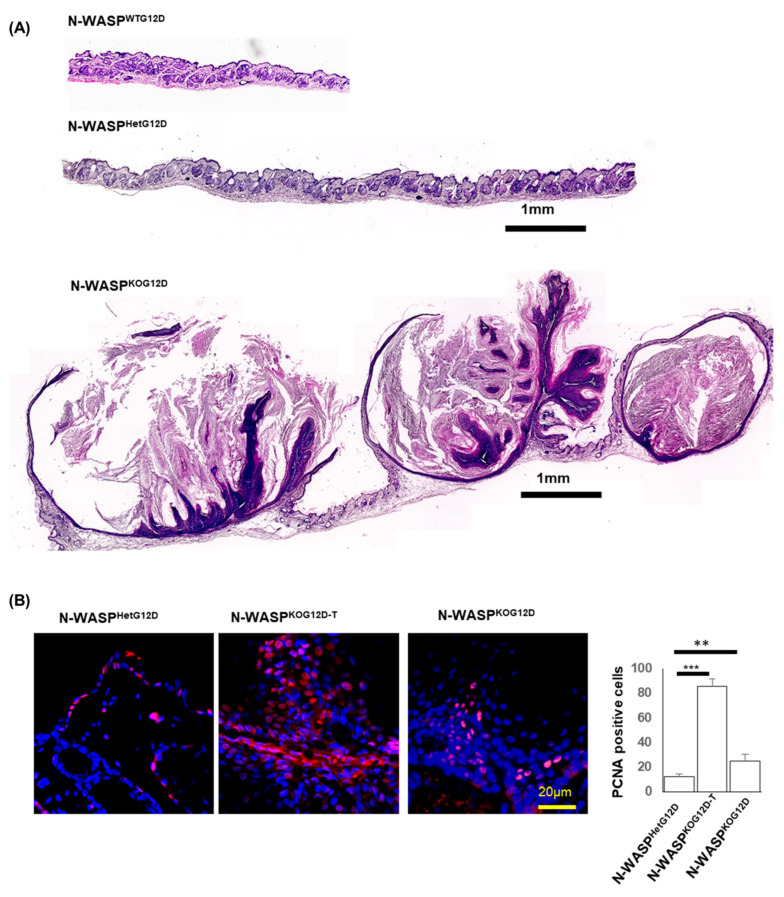
The ablation of N-WASP in KRas^G12D^-expressing cells lead to the formation of Squamous Cell Carcinoma (SCC). (**A**) H&E-stained dorsal skin sections of N-WASP^WTG12D^, N-WASP^HetG12D^, and N-WASP^KOG12D^ mice at 37 days post TAM injection (n = 11); (**B**) PCNA immunostaining of paraffin-embedded tumor dorsal skin sections of N-WASP^KOG12D^ and N-WASP^HetG12D^ mice at 37 days post TAM injection and the quantification of PCNA-positive cells (n = 3). The positive staining of PCNA in N-WASP^KOG12D^ and N-WASP^HetG12D^ skin sections were manually counted at random fields, as shown in the figure. Results: means ± SDs. *** *p* < 0.001; ** *p* < 0.01.

**Figure 5:**
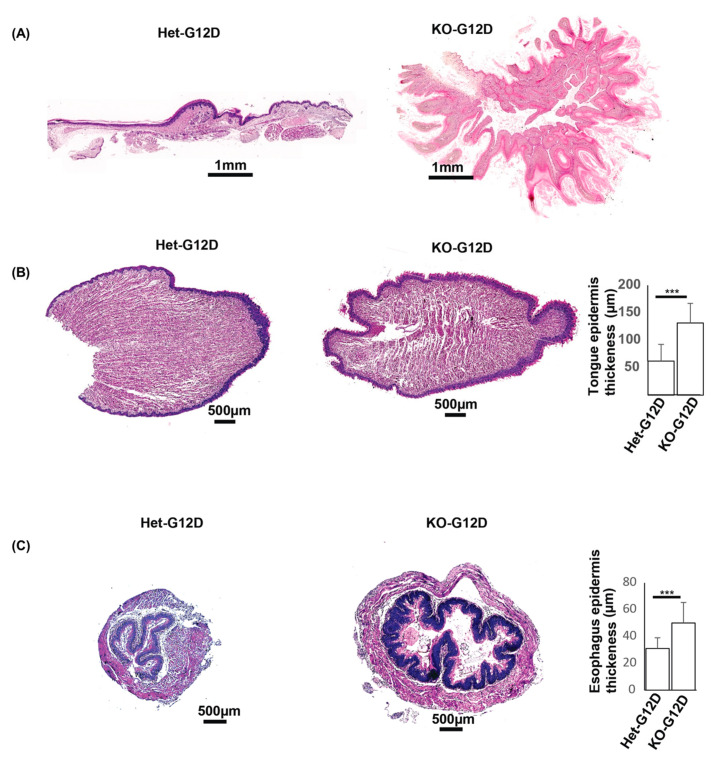
The ablation of N-WASP and expression of KRas^G12D^ induced epidermal thickening in the tongue, esophagus, and palm/soles. H&E-stained samples from the palm/soles (**A**), tongue (**B**), and esophagus (**C**) sections of the N-WASP^KOG12D^ and N-WASP^HetG12D^ mice at 37 days post TAM injection (n = 11). Quantification of epidermal thickening in the tongue and esophagus samples of N-WASP^KOG12D^ and N-WASP^HetG12D^ mice at 37 days post injection (n = 5). Results: means ± SDs. *** *p* < 0.001.

To shed light on the role of N-WASP in KRas^G12D^-induced cancer, H&E staining was performed in tumors from multiple sites from N-WASP^KOG12D^ and N-WASP^HetG12D^ mice, such as the dorsal skin, palms and soles, esophagus, and tongue, at 37 days following the TAM injections. The staining displayed poorly differentiated SCCs with an abundance of keratin pearls in the skin of the N-WASP^KOG12D^ mice, in opposition to that of the N-WASP^HetG12D^ mice (Figure 4A). Next, we performed PCNA immunostaining, which showed a significantly higher number of PCNA-positive cells in the tumor regions of the N-WASP^KOG12D^ mice, suggesting an increased rate of proliferation of the epidermal keratinocytes in these mice as compared to the N-WASP^HetG12D^ mice (Figure 4B). Similarly, we observed large tumors on the palms and soles of the N-WASP^KOG12D^ mice at 37 days post TAM which were not seen in N-WASP^HetG12D^ mice (Figure 5A).

Moreover, the epidermal layers of the tongue and esophagus from the N-WASP^KOG12D^ mice were found to be significantly thicker compared to those of the N-WASP^HetG12D^ mice at 37 days post TAM (Figure 5B,C). Previous reports clarified that SCCs are characterized by the expression of stratified epithelial keratins (K5, K14, and K17), focally expressed keratins (K1/K10), hyperproliferative keratinocyte keratins (K6 and K16) [30], and poorly differentiated squamous cell carcinoma keratin markers such as the keratins K8, K18, and K19 [31,32,33]. Our immunostaining revealed a high level of expression of an early skin carcinoma marker (K17) and poorly differentiated SCC keratin markers (K18 and K19) in the skin tumors of the N-WASP^KOG12D^ mice (Figure S4C–F). These results were further substantiated through a Western blot, with the epidermal protein lysate of the N-WASP^KOG12D^ mice exhibiting higher levels of expression of K10, K17, K18, and K19 in contrast with the lysate from the opposing group (Figure S4A,B). Thus, our results suggest that the loss of N-WASP in basal keratinocytes gives rise to abnormal keratin expression in the epidermis and accelerates the formation of poorly differentiated SCC in mice with KRas^G12D^-induced tumors.

### 3.5. The Loss of N-WASP in Mice with KRas^G12D^-Induced Tumors Activates the EGFR and KRas Pathways

**Figure 6:**
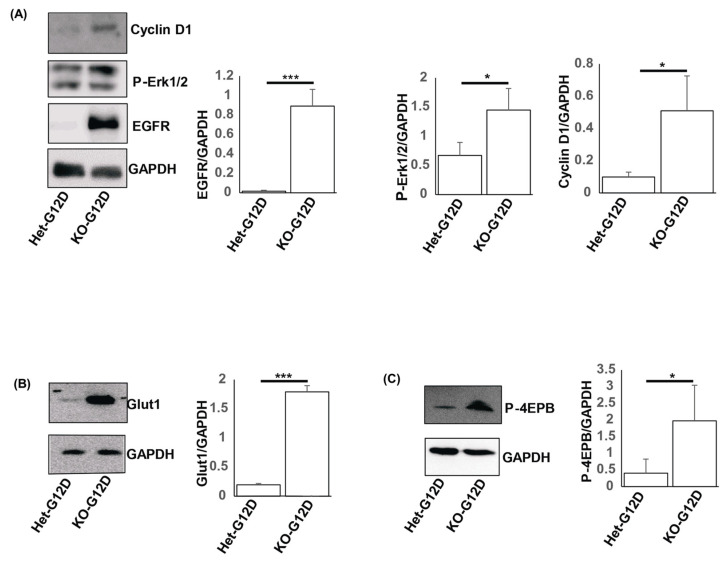
The loss of N-WASP in mice with KRas^G12D^-induced tumors activates the EGFR and KRas signaling pathway. Protein lysates from epidermis samples of the N-WASP^KOG12D^ and N-WASP^HetG12D^ mice (at 37 days post TAM injection) were subjected to a Western blot analysis, using antibodies against Cyclin D1, EGFR, P-ERK1/2 (**A**), Glut1 (**B**), and P-4EPB. (**C**) GAPDH was used as a loading control (n = 3). Results: means ± SEs. *** *p* < 0.001 and * *p* < 0.05. uncropped WB images were shown in Figure S8.

Prior studies outlined that hyperproliferation, the hyperkeratosis of the epidermis, and defects in the infundibula and SGs are due to the increased activation of EGFR and KRas signaling within the skin [34,35]. To investigate the molecular mechanism behind the accelerated skin tumor growth in the N-WASP^KOG12D^ mice, we isolated protein lysates (37 days post TAM) from the skin of both groups of mice and carried out Western blot analyses for EGFR, P-Erk, and Glut1 (Figure 6). 

We noticed that the expression levels of EGFR, P-Erk, and Glut1 were significantly higher in the skin of the N-WASP^KOG12D^ mice, implying that the deletion of N-WASP in the epidermal keratinocytes of these mice led to the dysregulation of the EGFR and Ras signaling pathway. These findings are congruent with previous studies in which the EGFR-Ras-Raf pathway was shown to play a crucial role in the progression of non-melanoma skin cancers, including SCC [36,37]. 

The rapid growth of a tumor requires a high amount of glucose uptake to support an increased metabolism. Considering this, we set out to ascertain the expression of the glucose transporters Glut1 and Glut3 in the skin of the N-WASP^HetG12D^ and N-WASP^KOG12D^ mice [38]. We found the enhanced expression of Glut1 in the skin of the N-WASP^KOG12D^ mice in comparison with the N-WASP^HetG12D^ mice. In addition, our Western blot and immunostaining results revealed significantly higher expression levels of PDGFR and SMA in the N-WASP^KOG12D^ mice when compared to the other group of mice, indicating the dysregulation of autocrine and paracrine signaling in the N-WASP^KOG12D^ mice (Figure S5A,B). In summary, our results show that the ablation of N-WASP expression accelerates KRas-mutant-induced tumor formation through the activation of the EGFR-KRas signaling pathway. 

Immune cell infiltration is a common feature of various solid tumors which has both prognostic value as well as therapeutic implications for tumor treatment. Several reports have shown that the infiltration of immune cells, such as the infiltration of macrophages in human breast cancer [39] and the infiltration of mast cells in melanoma, correlates with poor patient prognosis [40]. We observed through Toluidine blue staining that there was an increased infiltration of mast cells in the N-WASP^KOG12D^ mice when compared to the N-WASP^HetG12D^ mice (Figure S6A). The role of mast cells in cancer remains unclear as they have been reported to play both pro-tumorigenic as well as anti-tumorigenic roles in cancer [41,42]. Enhanced tumor progression was previously attributed to higher expression levels of chemokines and cytokines in solid tumor microenvironments which consequently attract and recruit neutrophils/macrophages to the tumor site [43]. Therefore, we proceeded to conduct immunostaining on the skin sections using ly6G/ly6C, a prominent marker for neutrophils/macrophages. The number of ly6G/ly6C-positive cells was found to be markedly higher in the N-WASP^KOG12D^ mice compared to their counterparts (Figure S6B). Hence, our results put forth that the infiltration of mast cells and neutrophils in the tumor microenvironment within the skin of the N-WASP^KOG12D^ mice potentially accelerates the formation and progression of tumors. 

## 4. Discussion

Squamous cell carcinoma (SCC) is the most common epithelial skin cancer and is driven by mutations in genes, such as the KRas and P53 genes [44]. As mutations in RAS account for 30% of human cancer and the expression of KRas^G12D^ in epidermal keratinocytes induces SCC formation in the skin [44], KRas^G12D^ mouse models are thus widely used to discern the role of KRas in the development of cancer [45,46]. The activation of KRas in the skin of mice induced changes in skin development and homeostasis, such as redundant skin, hair loss, perianal papillomas, and the formation of SCC in Msx2Cre; KRas^G12D^ mice [35]. The inducible LSL-KRas^G12D^ mouse model has been commonly used to identify the roles of tumor suppressors or oncogenic drivers in the formation and progression of tumors [45]. N-WASP, a member of the WASP family of proteins, is expressed ubiquitously and regulates actin polymerization via the modulation of the activity of the Arp2/3 complex [8]. The global knockout of N-WASP in mice leads to embryonic lethality, highlighting that N-WASP is integral to early embryonic development [47]. Alternatively, the conditional knockout of N-WASP in muscle using Myf5-cre and MyoD-cre [48] and in the brain using Nestin-cre [49] caused postnatal lethality. Recently, we determined that loss of N-WASP in epidermal keratinocytes led to epidermal thickening, barrier defects, immune cell infiltration, and an atopic-dermatitis-like inflammation of the skin [9]. 

Earlier reports demonstrated that higher levels of expression of N-WASP in pancreatic adenocarcinoma [16], liver adenocarcinoma [17] and breast carcinoma [18] are correlated with the poor survival of patients. On the other hand, numerous studies have shown that the reduced levels of expression of N-WASP in colorectal cancer [23], Stage 1 lung cancer [19], and renal cancer [21] are associated with poor prognosis and the reduced survival of patients. However, the role of N-WASP in epithelial skin cancer has yet to be defined. To amply comprehend the role of N-WASP in skin SCC, we generated a TAM-inducible mouse model that allowed for the activation of the dominantly active KRas^G12D^ mutant expression, coupled with either the homozygous (N-WASP^KOG12D^) or heterozygous (N-WASP^HetG12D^) loss of N-WASP in the epidermis of the skin, driven by keratin 5 (K5-CreERT). 

Our results reveal that in comparison to the N-WASP^HetG12D^ mice, tumors appeared in the non-hair-bearing areas on the N-WASP^KOG12D^ mice, such as the lips, dorsal skin, anus, palm/soles, eyelids, and ears, as early as 2 weeks post TAM injection. We found that the N-WASP^KOG12D^ mice developed skin tumors with an incidence of 100% and survived until Day 48, while the N-WASP^HetG12D^ mice survived up to 116 days. Hence, it appears that the deletion of N-WASP in basal epithelial cells accelerated the formation and growth of tumors induced by KRas^G12D^.

Within 37 days of the injections, we observed the development of acanthosis, the keratinization of the infundibula, dilated infundibula, and keratin cysts of an array of sizes with the absence of SGs or small SGs in the skin of the N-WASP^KOG12D^ mice, while the N-WASP^HetG12D^ mice possessed enlarged SGs with no tumors along the dorsal skin. Several reports have suggested that the formation of an abnormal pilosebaceous unit, hyperkeratosis, and keratin cysts in mice may be ascribed to the activation of EGFR signaling and the Nrf2-induced upregulation of Epgn [26]. We found that at 37 days post TAM, there were no significant changes in the expression of Nrf2 and its target gene Epgn in the N-WASP^KOG12D^ mice, while expression of EGFR was notably higher in comparison with the N-WASP^HetG12D^ mice. As such, our results show that the upregulated production of keratins, the formation of cysts, and the SG abnormalities were caused by abnormal signaling within the EGFR pathway. EGFR has been shown to induce an alteration in the AhR/ARNT signaling pathway, leading to SG abnormality. The pharmacological inhibition of EGFR, however, reduces SG abnormalities in the skin [50]. 

Moreover, a previous study illustrated that the upregulation of SPrr2d in K5cre-CMVcaNrf2 mice correlated with acanthosis of the infundibula and the subsequent disruption of the follicular barrier. Therefore, we scrutinized the expression levels of Slpi and Sprr2d at 37 days post TAM injection and found the expression of Slpi to be significantly higher in the N-WASP^KOG12D^ mice compared to the N-WASP^HetG12D^ mice. At this timepoint, we also observed scores of keratin-filled cysts of different sizes on the N-WASP^KOG12D^ mice which were not seen on the skin of the latter group. This phenotype may be associated with the upregulation of Slpi and Sprr2d in the epidermis and infundibula, which led to the progressive overproduction of keratin in the infundibula and the formation of cutaneous keratin cysts [26,51]. Surprisingly, the characteristic features of the N-WASP^KOG12D^ mice, such as a reduction in SGs or no SGs and the formation of cutaneous keratin cysts, can be correlated with the pathology of MADISH (metabolizing acquired dioxin-induced skin hamartomas) patients [26]. The increased expression levels of Slpi, Sprr2d, Nqo1, and Epgn in the affected epidermis and cutaneous cysts of a MADISH patient’s skin are reportedly mediated via follicular hyperplasia and hyperkeratosis, as evidenced by a prior study [26]. 

Keratins are the intermediate filaments of epithelial cells; they are differentially expressed in epithelial cells and can be used as potential markers to identify the origin of SCC [52]. Moll et al., 2008 stated that keratin pearls are associated with K8, K18, and K19, which are expressed in poorly differentiated SCC [30]. Our results highlighted a significantly enhanced expression of several differently sized keratin pearls with K8, K18, K17, and K19 filaments within the dorsal skin tumors of the N-WASP^KOG12D^ mice. Subsequently, our findings also emphasized that the absence of N-WASP in the basal epithelium of the skin accelerated the formation of SCC in the KRas^G12D^-induced mice in comparison with their control counterparts. 

Next, we observed significantly upregulated levels of expression of PDGFR and SMA in the skin of the N-WASP^KOG12D^ mice as compared to those of the N-WASP^HetG12D^ mice, indicating that autocrine and paracrine signaling may be involved in tumor progression. Furthermore, we found that EGFR, Glut1, and p-ERK2 were substantially higher in N-WASP^KOG12D^ mice, suggesting that the ablation of N-WASP expression activates the EGFR/MAPK pathway in mice expressing KRas^G12D^. Glut-1 expression is considered critical to glucose uptake during neoplastic transformation [38], and we found that the N-WASP^KOG12D^ mice had a higher level of expression of Glut1 compared to the N-WASP^HetG12D^ mice. Previous studies stated that the activation of KRas correlated with higher levels of expression of EGFR/MAPK in a plethora of human cancers [46,53]. Furthermore, N-WASP has been shown to be essential to the clathrin-mediated endocytosis of receptors such as EGFR, and thus the higher level of expression of EGFR in tje N-WASP^KOG12D^ mice could be due to a reduction in endocytosis [54]. 

## 5. Conclusions

In summary, we demonstrated that the deletion of N-WASP enhanced the formation of tumors in mice with KRas^G12D^-induced tumors through the activation of the EGFR and KRas signaling pathway. Our results further defined N-WASP as a tumor suppressor in KRas^G12D^-induced skin cancer, and it could be a novel biomarker for skin SCC.

## Figures and Tables

**Table 1:** Incidence of tumors in the various anatomical regions of mice.

Anatomical Regions of Mice	Incidence of Tumors in Male Mice	
	Mice with Tumors/Total Number of Mice	
	Homo	Het
Dorsal skin	9/15	0/12
Anus	15/15	12/12
Face	13/15	5/12
Palm/soles	15/15	5/12
Ears	2/15	1/12
Lips	8/15	6/12
Eyelids	8/15	6/12
Stomach	15/15	8/12
Colon	3/15	0/12

## Data Availability

The data presented in this study are available within the article and Supplementary Materials.

## Supplementary Materials

The following supporting information can be downloaded at: https://www.mdpi.com/article/10.3390/cancers15184455/s1, Figure S1: Generation of homozygous and heterozygous N-WASP knockout mice expressing KRas^G12D^; Figure S2: KRas^G12D^ accelerate tumor growth in N-WASP^KOG12D^ mice compared to N-WASP^HetG12D^ mice; Figure S3: Keratin cyst formed in the dorsal skin of N-WASP^KOG12D^ mice 21 days after TAM injection; Figure S4: Expression levels of PTP63, K19, and Cyclin B1 are increased in N-WASP^KOG12D^ mice; Figure S5: Expression levels of SMA and PDGFR are increased in the dorsal skin of N-WASP^KOG12D^ mice; Figure S6: Increased infiltration of neutrophils and mast cells in the dorsal skin of N-WASP in KRas^G12D^; Figure S7: Ablation led to a decrease in the expression of transglutaminase and involucrin in N-WASP^KOG12D^ mice compared to N-WASP^HetG12D^ mice. Figure S8: uncropped WB images.
