# Supplementary material for: Conditional Knockout of N-WASP Enhanced the Formation of Keratinizing Squamous Cell Carcinoma Induced by KRasG12D
Source: Cancers (Basel). 2023 Sep 7;15(18):4455. doi: 10.3390/cancers15184455 (PMC10526518; doi:10.3390/cancers15184455)
Supplement: Supplementary file 1 [file cancers-15-04455-s001.zip › cancers-2531743-supplementary.pdf]

Figure legends:

Fig.S1

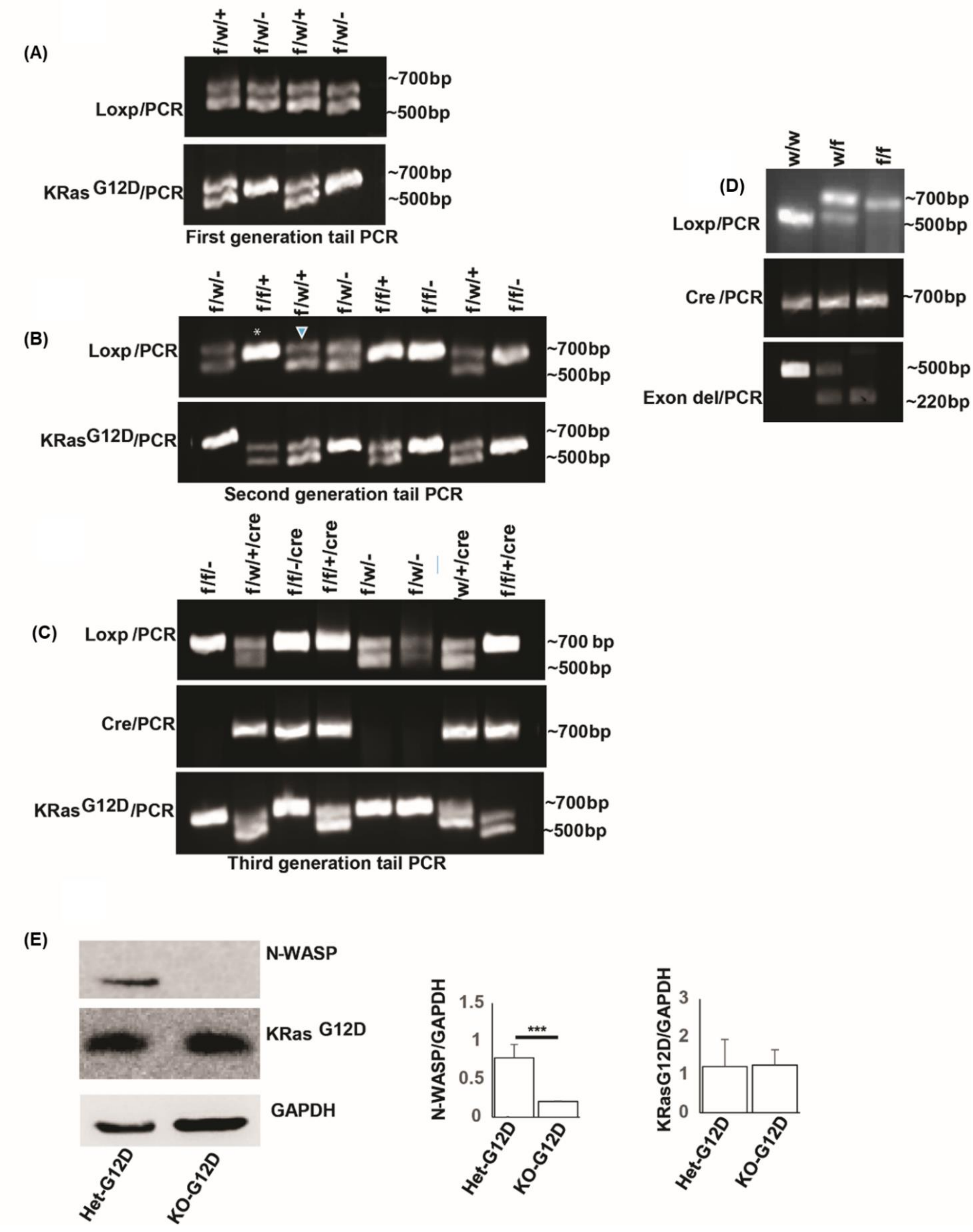

Figure S1. Generation of homozygous and heterozygous N-WASP knockout mice expressing $KRas^{G12D}$. (**A**) PCR of tail genomic DNA from 1st generation $N\text{-}WASP^{WT/fl}$ mice, (700 bp: floxed allele, 500 bp: WT allele); (**B**) PCR of tail genomic DNA from mice derived from a second generation mating to produce *N-WASP*$^{fl/fl}$; *KRas*^{G12D}. (700bp: WT allele, 500bp: Loxp-Stop-Loxp-$Kras^{G12D}$ allele); (**C**) PCR genotyping of mice from third generation mating, using tail genomic DNA (*N-WASP*$^{fl/fl}$; *KRas*^{G12D} X *N-WASP*$^{WT/fl}$; *K5-Cre*). Asterisk (*) indicates homozygous, *N-WASP*$^{fl/fl}$; *KRas*^{G12D}; *K5-Cre* mice, (↑) indicates heterozygous, *N-WASP*$^{WT/fl}$; *KRas*^{G12D}; *K5-Cre* mice; (**D**) PCR of tail genomic DNA showing the product of exon 3 and 4 deletion within the N-WASP gene; (**E**) Protein lysates from the epidermis of $N\text{-}WASP^{KOG12D}$ and $N\text{-}WASP^{HetG12D}$ mice were subjected to western blot, using antibodies against N-WASP, *KRas*^{G12D} and GAPDH (loading control) (n = 3). Results are mean ± SD (***$p < 0.001$).

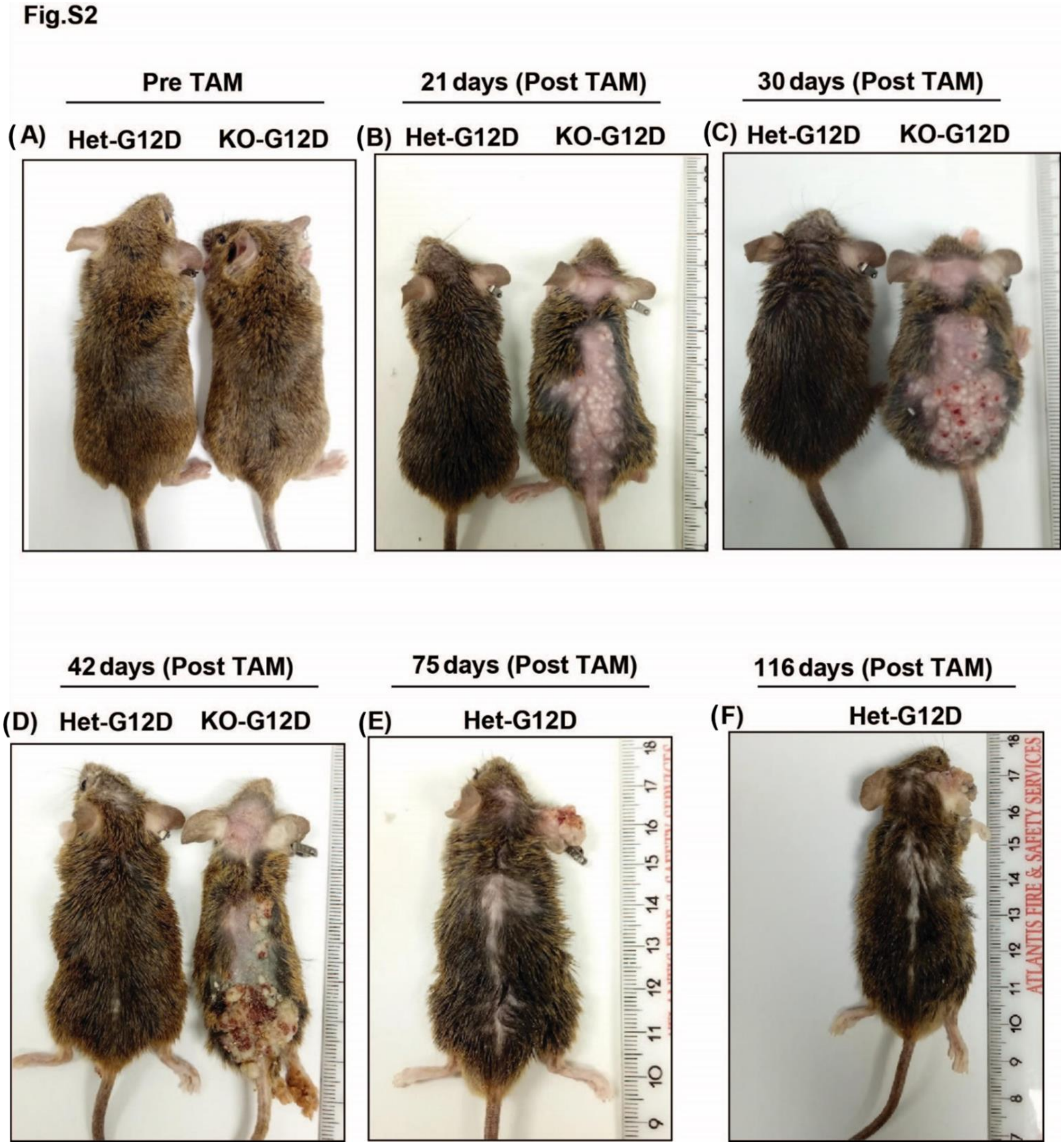

Figure S2. Homozygous knockout of N-WASP accelerates growth of KRas^{G12D} induced tumors. Tumors appeared earlier on the dorsal skin of the N-WASP^{KOG12D} mice (12 days), as opposed to the N-WASP^{HetG12D} mice. The tumors on the former group progressed rapidly (21st, 30th and 42nd day) and all the N-

WASP^{KOG12D} mice died within 48 days of the TAM injections (**A–D**). Heterozygous mice survived up to 116 days, with tumors only visible on the face, eyelid, palm/sole (**E** & **F**).

Fig.S3

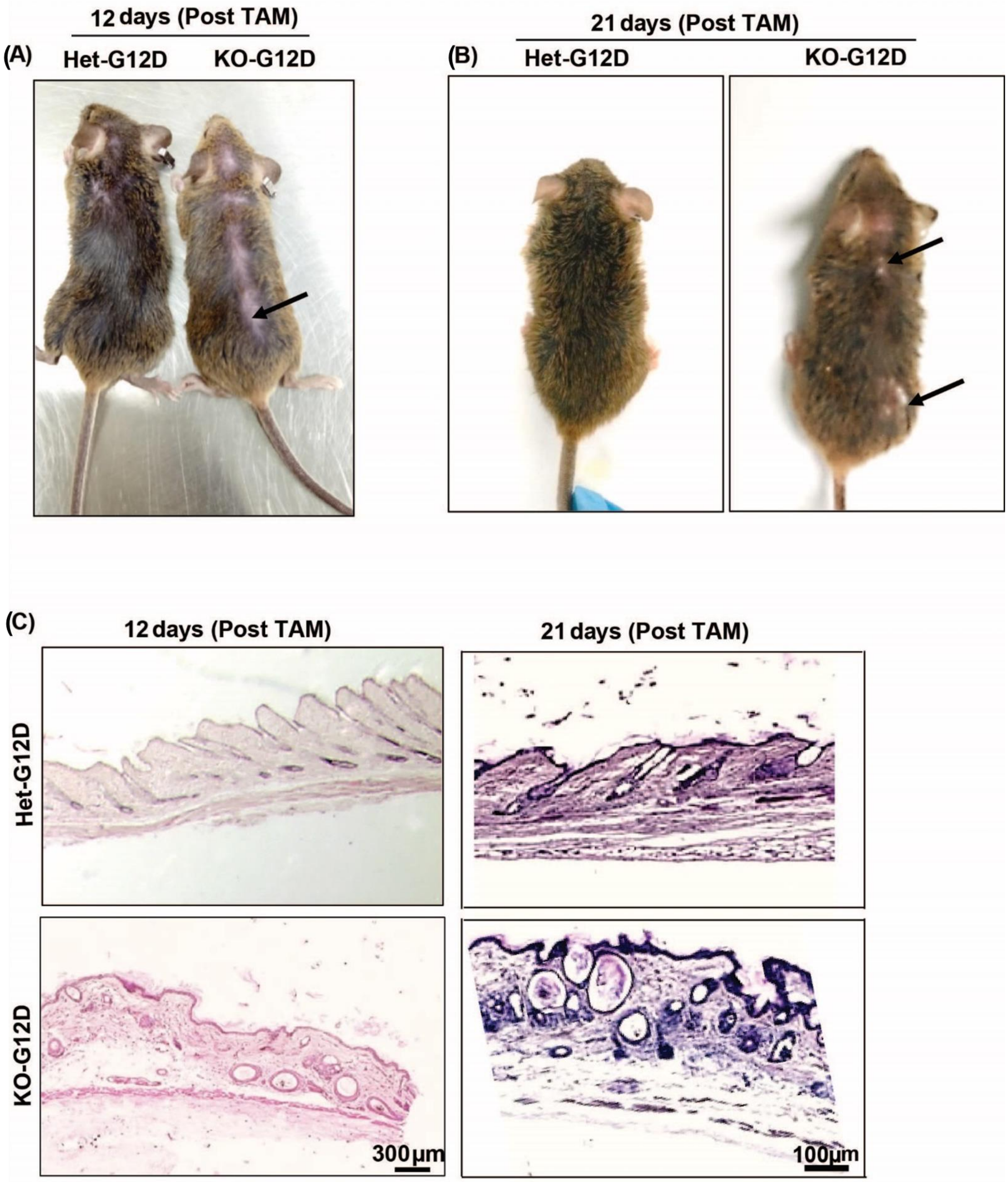

12 days (Post TAM)
(A)
Het-G12D
KO-G12D
21 days (Post TAM)
(B)
Het-G12D
KO-G12D
(C)
12 days (Post TAM)
21 days (Post TAM)
Het-G12D
KO-G12D
300 µm
100µm

Figure S3. Keratin cysts formed on dorsal skin of N-WASP^{KOG12D} mice 21 days after TAM injection. Small papillomas (arrow) appeared on the N-WASP^{KOG12D} mice at days 12 and 21 post TAM (**A** & **B**). H & E-stained dorsal skin sections from both mice groups, 21 days post-injection (n = 3) (**C**).

Fig.S4

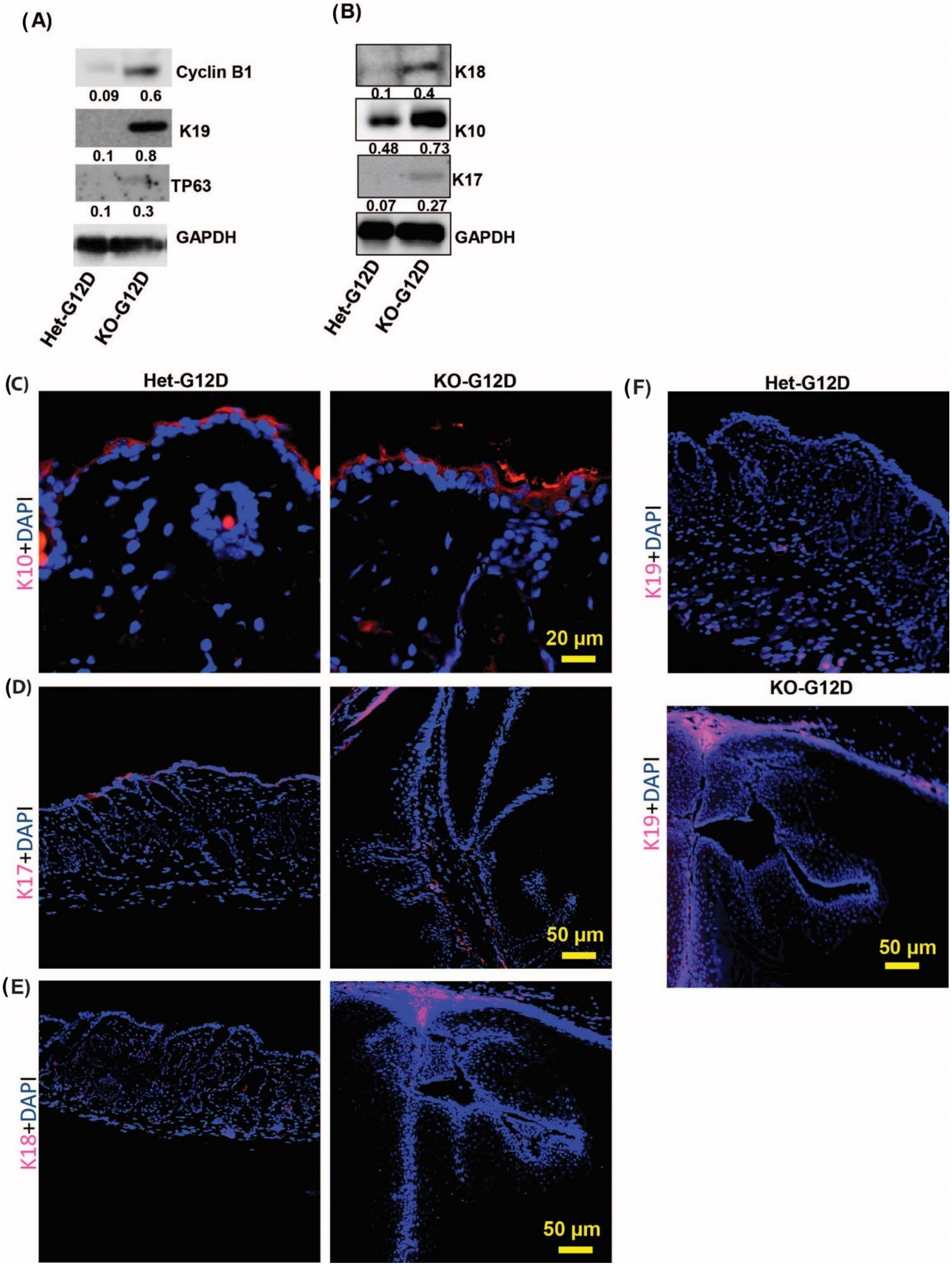

(A)
Cyclin B1
0.09 0.6
K19
0.1 0.8
TP63
0.1 0.3
GAPDH
Het-G12D
KO-G12D
(B)
K18
0.1 0.4
K10
0.48 0.73
K17
0.07 0.27
GAPDH
Het-G12D
KO-G12D
(C)
Het-G12D
KO-G12D
K10+DAPI
20 µm
(D)
K17+DAPI
50 µm
(E)
K18+DAPI
50 µm
(F)
Het-G12D
K19+DAPI
KO-G12D
K19+DAPI
50 µm

Figure S4. Expression of TP63, K19 and Cyclin B1 is increased in N-WASP^{KOG12D} mice. Protein lysates from the epidermis of N-WASP^{KOG12D} and N-WASP^{HetG12D} mice were subjected to western blot, using antibodies against TP63, K19 and Cyclin B1 (**A**), K10, K17, K18 (**B**). GAPDH was used as a loading control (n = 3). Immunostaining of K10 (**C**), K17 (**D**), K18 (**E**) and K19 (**F**) in paraffin-embedded skin tissue of the N-WASP^{KOG12D} and N-WASP^{HetG12D} mice 7 weeks post TAM injection (n = 3).

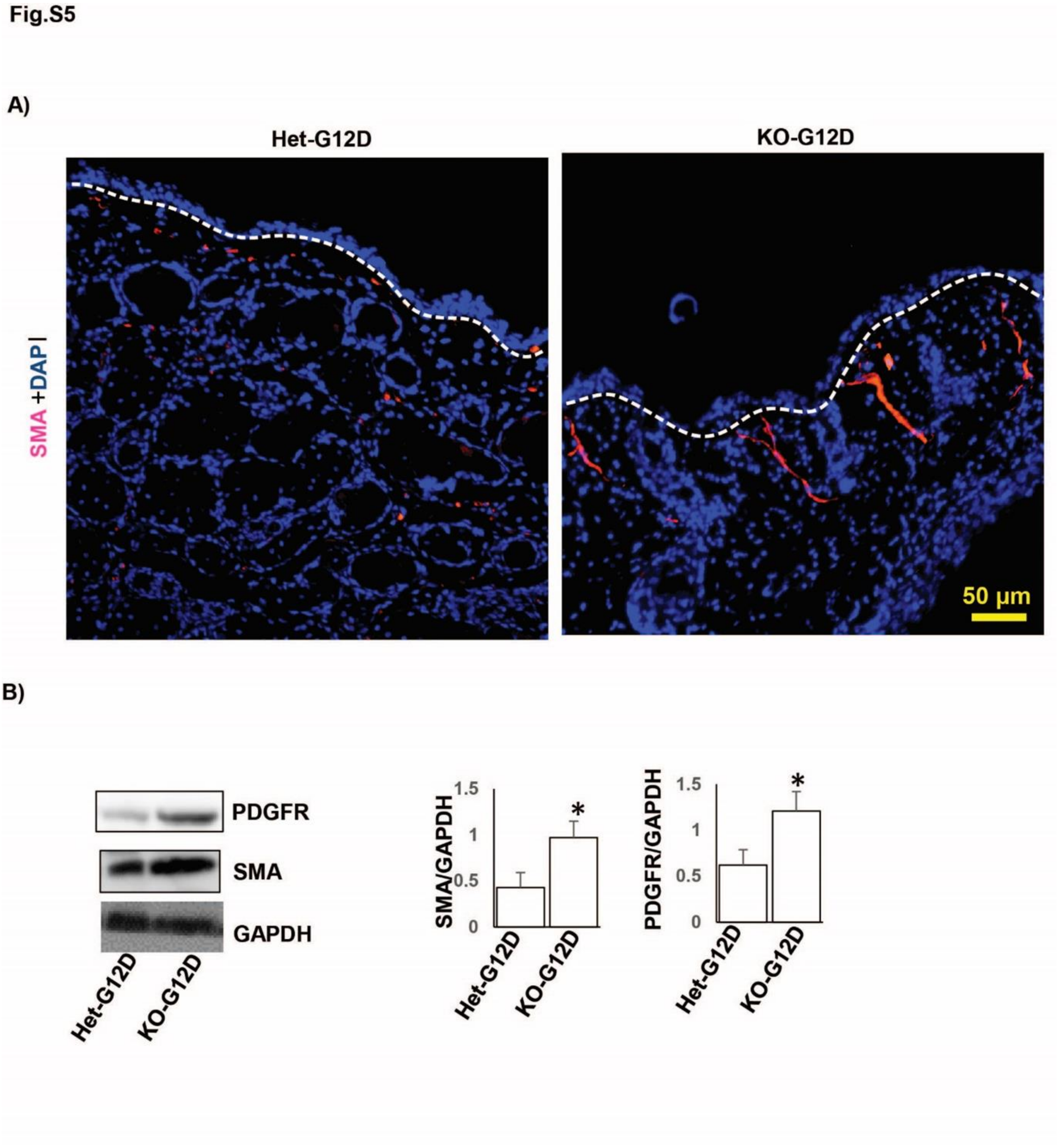

Figure S5. Expression of SMA and PDGFR is increased in the dorsal skin of N-WASP^{KOG12D} mice. (**A**) Immunostaining of SMA in paraffin-embedded skin tissue of N-WASP^{KOG12D} and N-WASP^{HetG12D} mice 7 weeks post TAM injection (n = 3). (B) Protein lysates from the epidermis of N-WASP^{KOG12D} and N-WASP^{HetG12D} mice were subjected to western blot analysis using antibodies against SMA and PDGFR. GAPDH was used as a loading control (n = 3). Results are mean ± SE * $p < 0.05$.

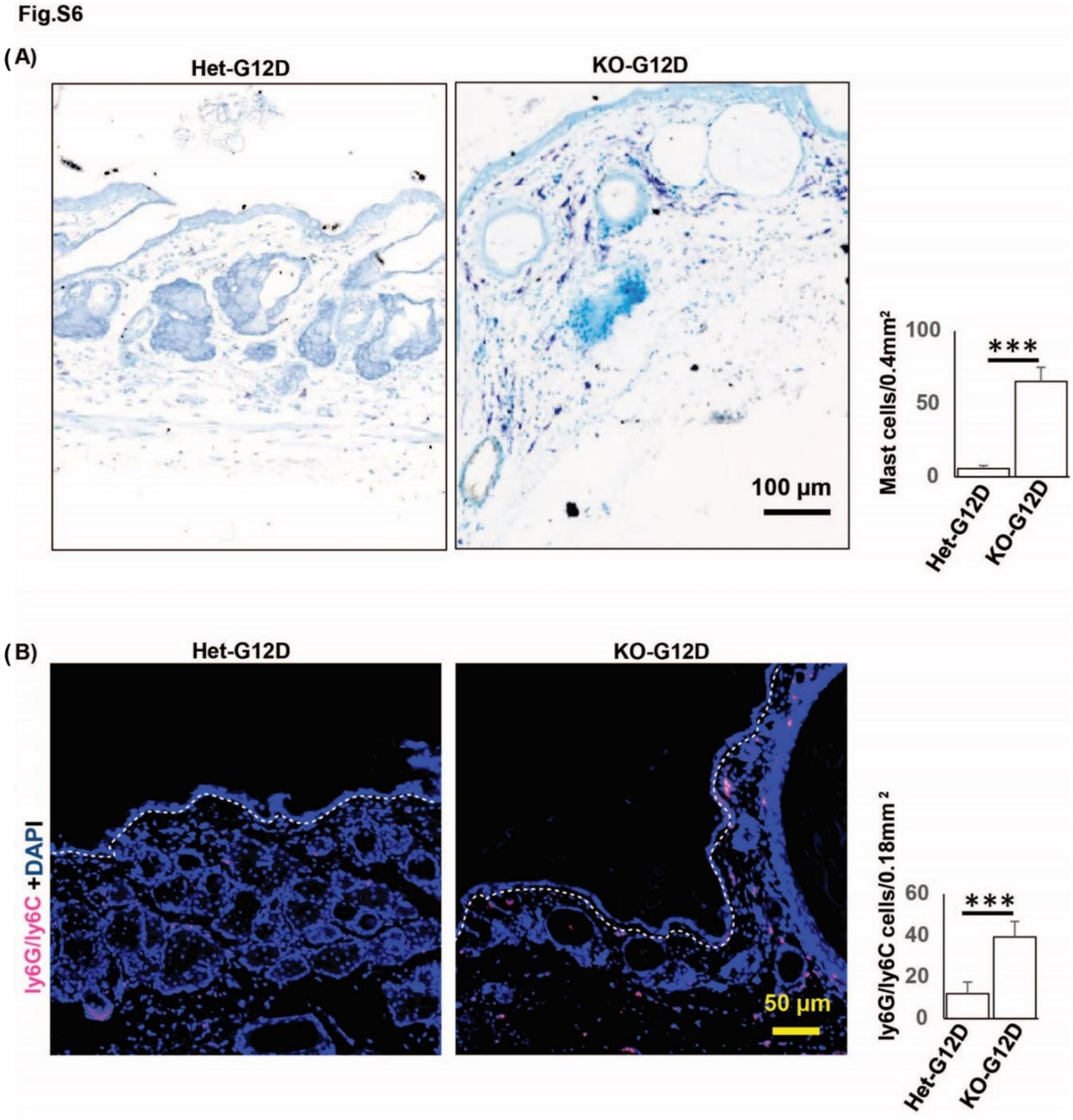

Figure S6. Increased infiltration of neutrophils and mast cells in dorsal skin of N-WASP^{KOG12D} mice. (A) Toluidine blue staining showed significant upregulation in infiltration of mast cells within the dermis of the skin of N-WASP^{KOG12D} mice, as compared to that of N-WASP^{HetG12D} mice 7 weeks post TAM injection (n = 3). (**B**) Immunostaining of Ly6G\6C in paraffin-embedded skin tissue of N-WASP^{KOG12D} and N-WASP^{HetG12D} mice 7 weeks post TAM injection (n = 3). Results are mean ± SE *** $p < 0.001$.

Fig.S7

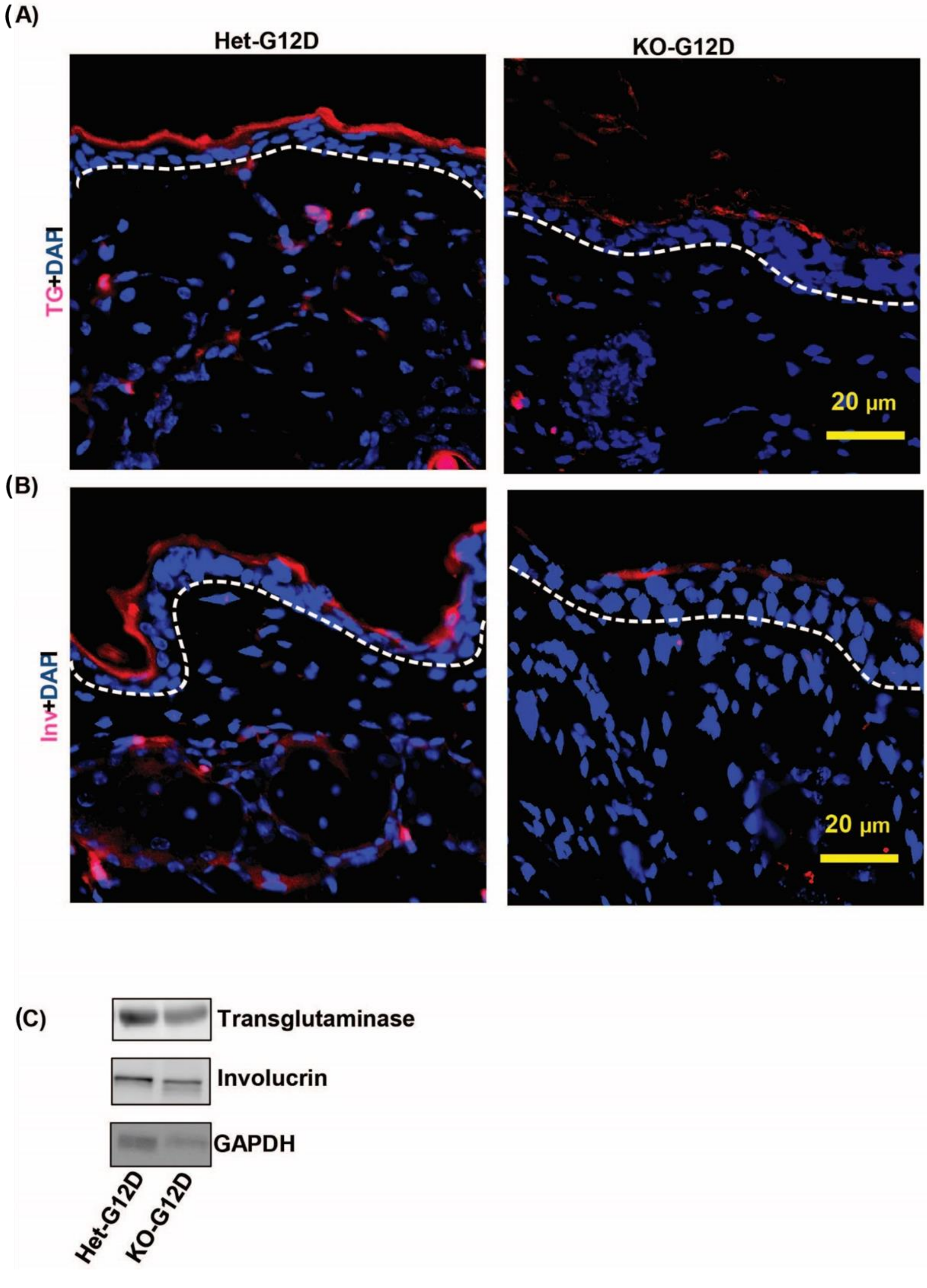
(A)
Het-G12D
KO-G12D
TG+DAPI
20 µm
(B)
Inv+DAPI
20 µm
(C)
Transglutaminase
Involucrin
GAPDH
Het-G12D
KO-G12D

Figure S7. Decreased expression of transglutaminase and involucrin in N-WASP^{KOG12D} mice as compared to N-WASP^{HetG12D} mice. Immunostaining of transglutaminase (**A**) and involucrin (**B**) in paraffin-embedded skin tissue of N-WASP^{KOG12D} and N-WASP^{HetG12D} mice 7 weeks post TAM injection (n = 3). (**C**) Protein lysates from the epidermis of N-WASP^{KOG12D} and N-WASP^{HetG12D} mice were subjected to western blot using antibodies against SMA and PDGFR. GAPDH was used as a loading control (n = 3).

Fig.S8

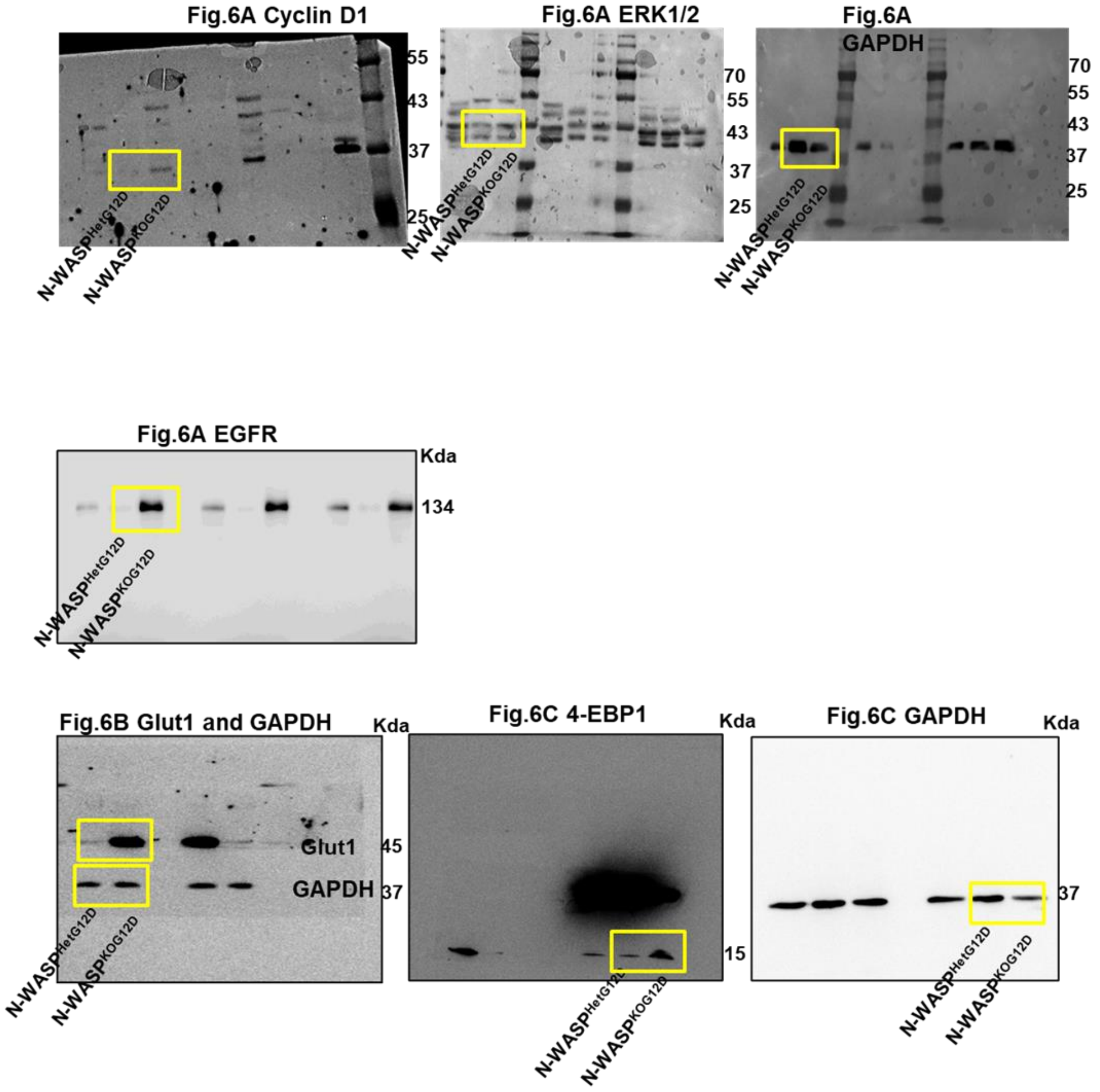

Figure S8. Uncropped Western blot images

Fig.S1E

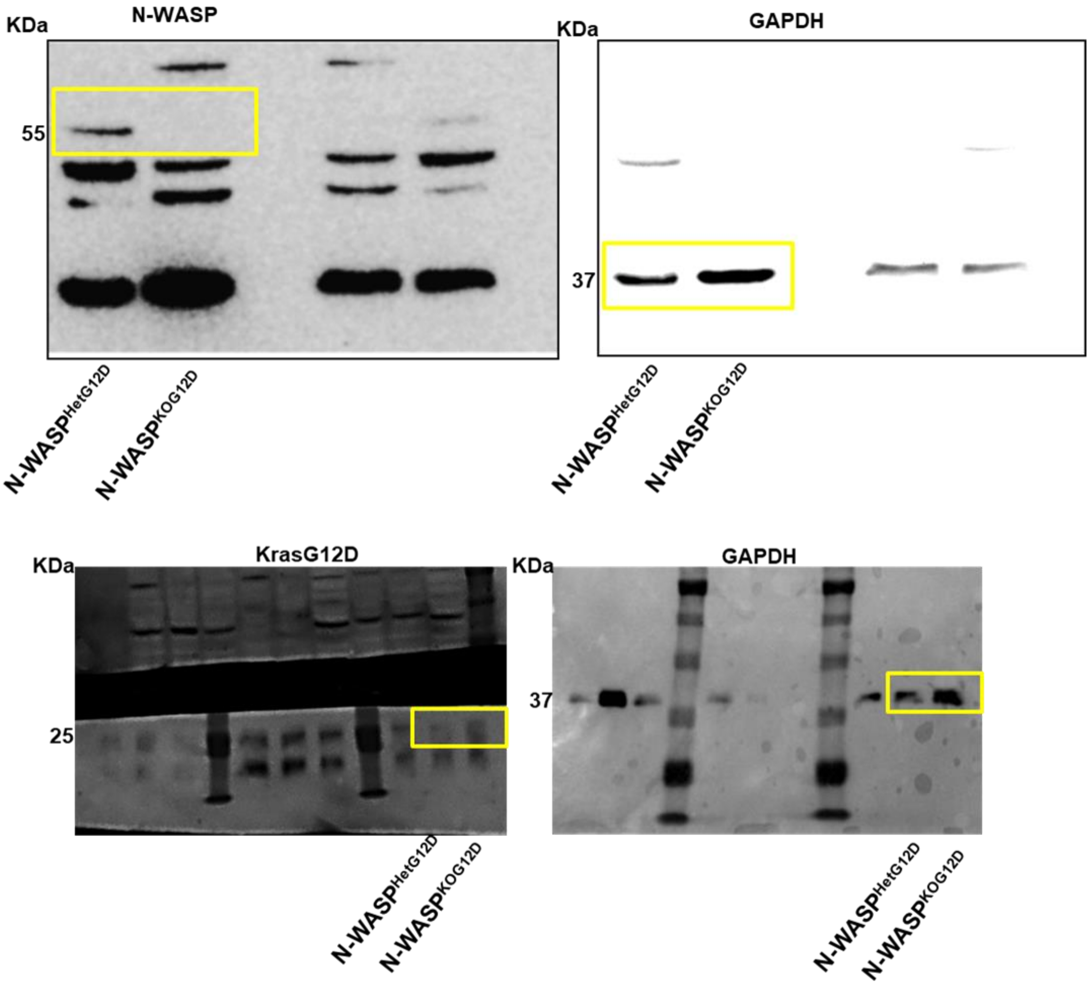

Figure S1E. Uncropped Western blot images

Fig.S4 A

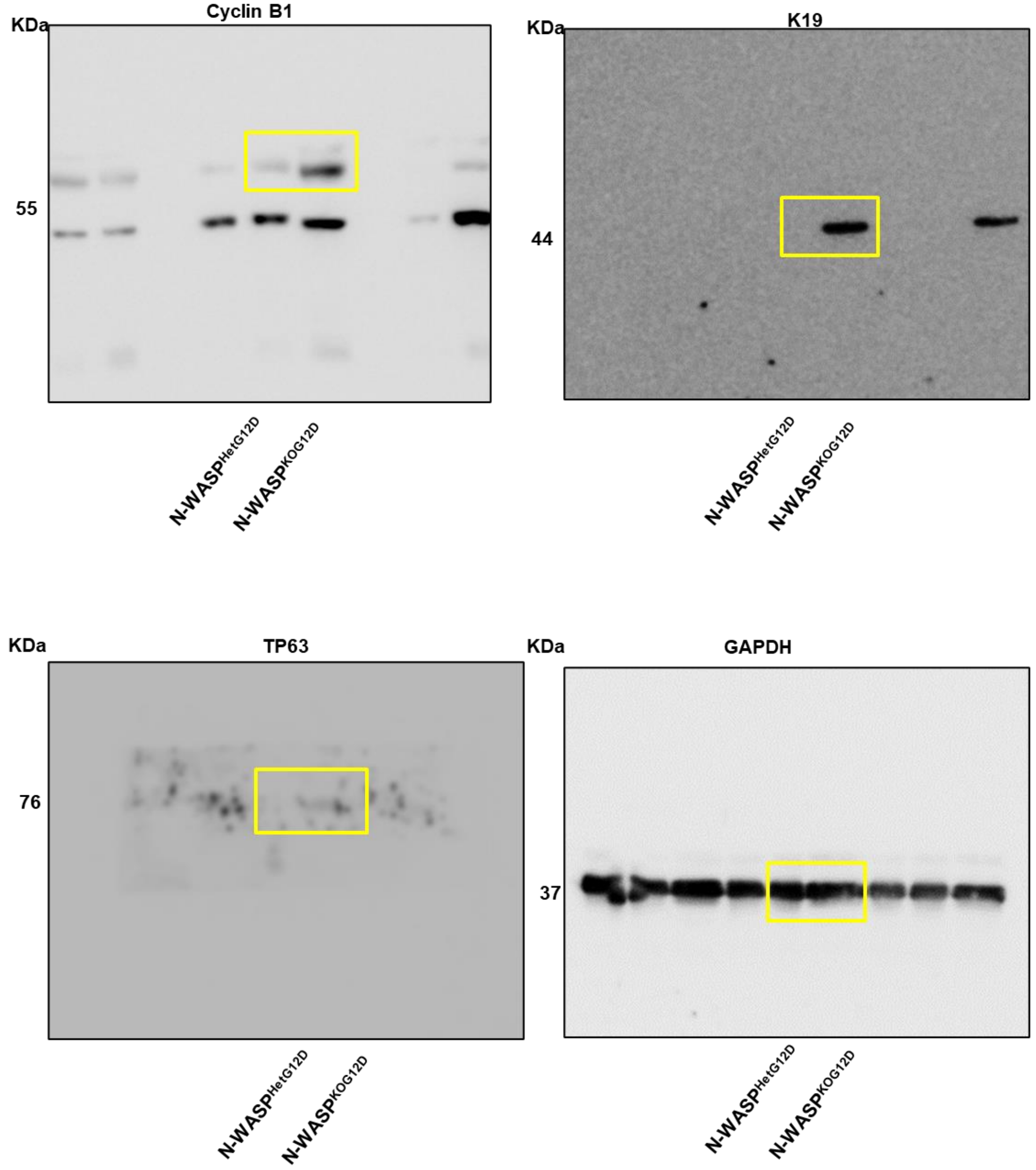

Figure S4A. Uncropped Western blot images

Fig.S4 B

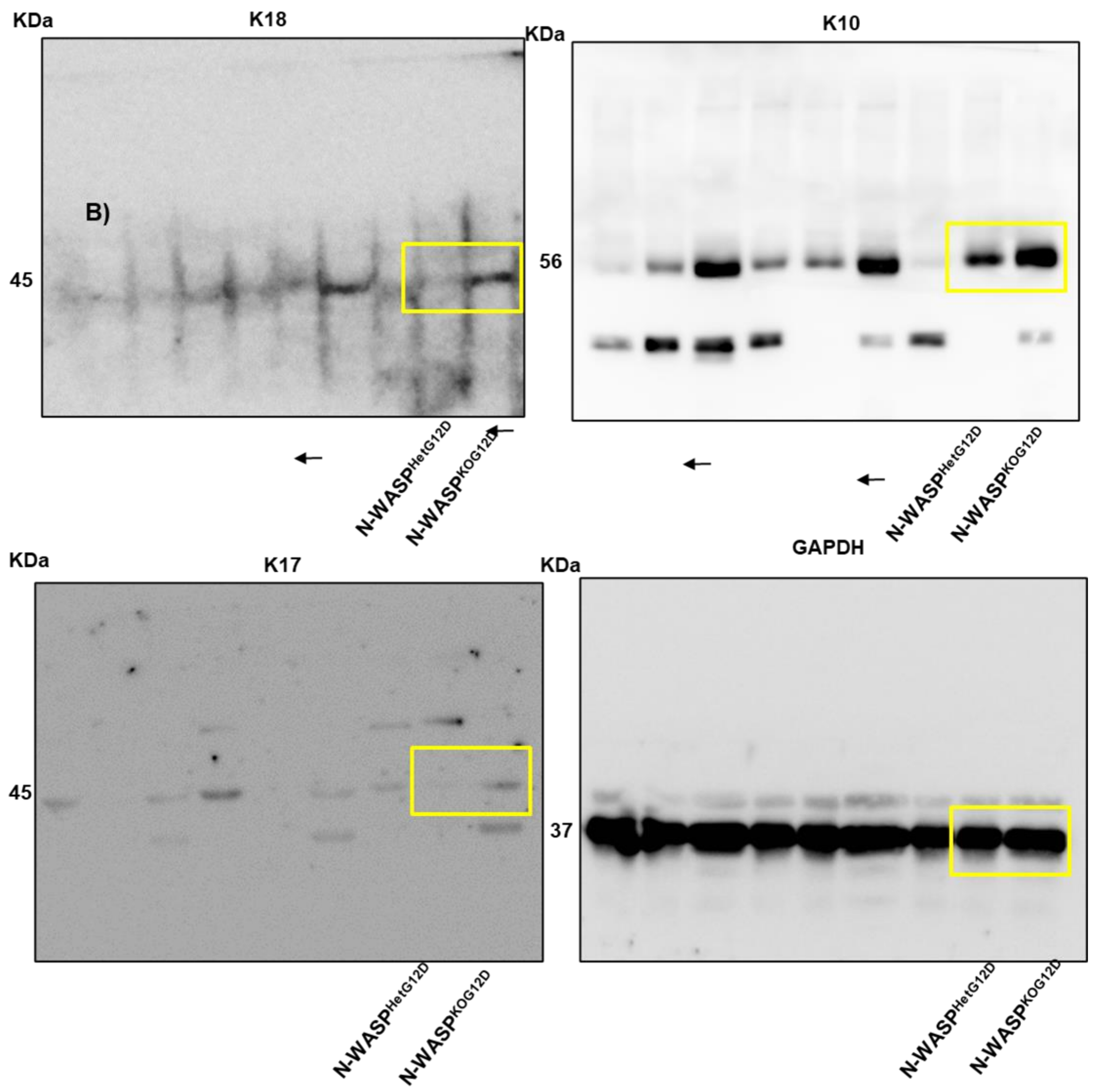

Figure S4B. Uncropped Western blot images

Fig.S5

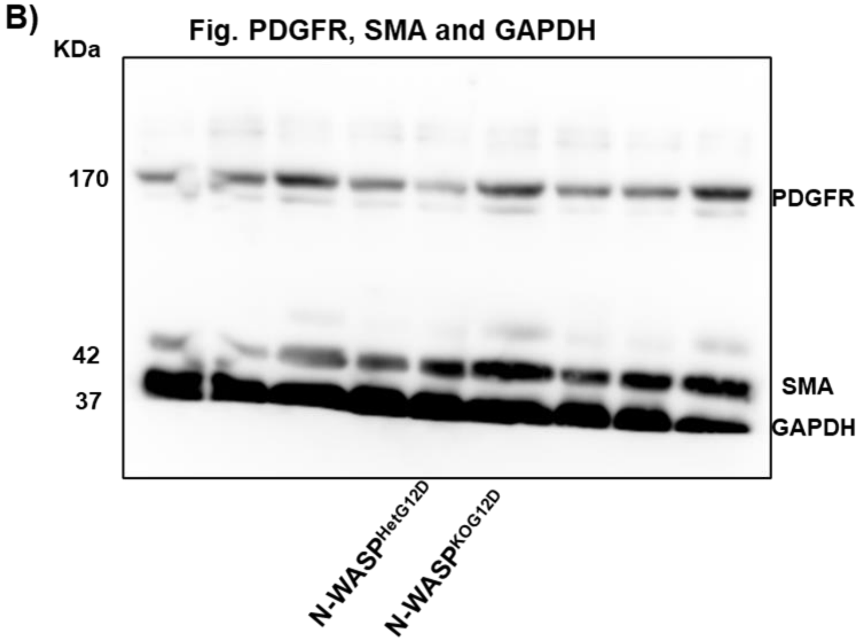

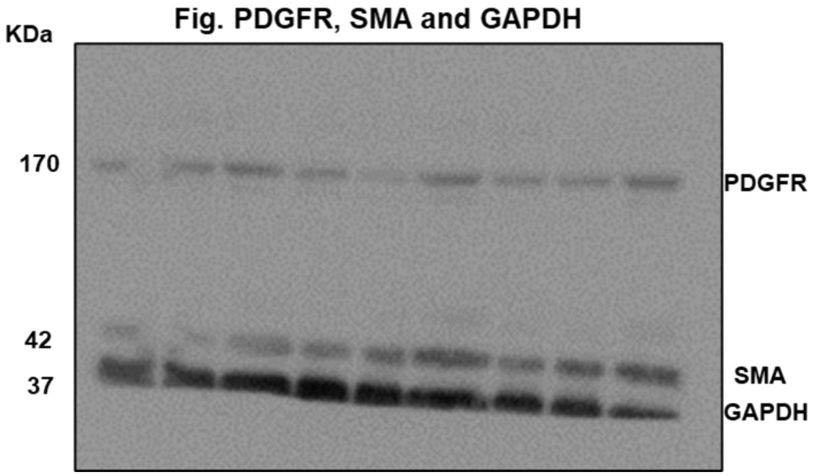

Figure S5B. Uncropped Western blot images

Fig.S7

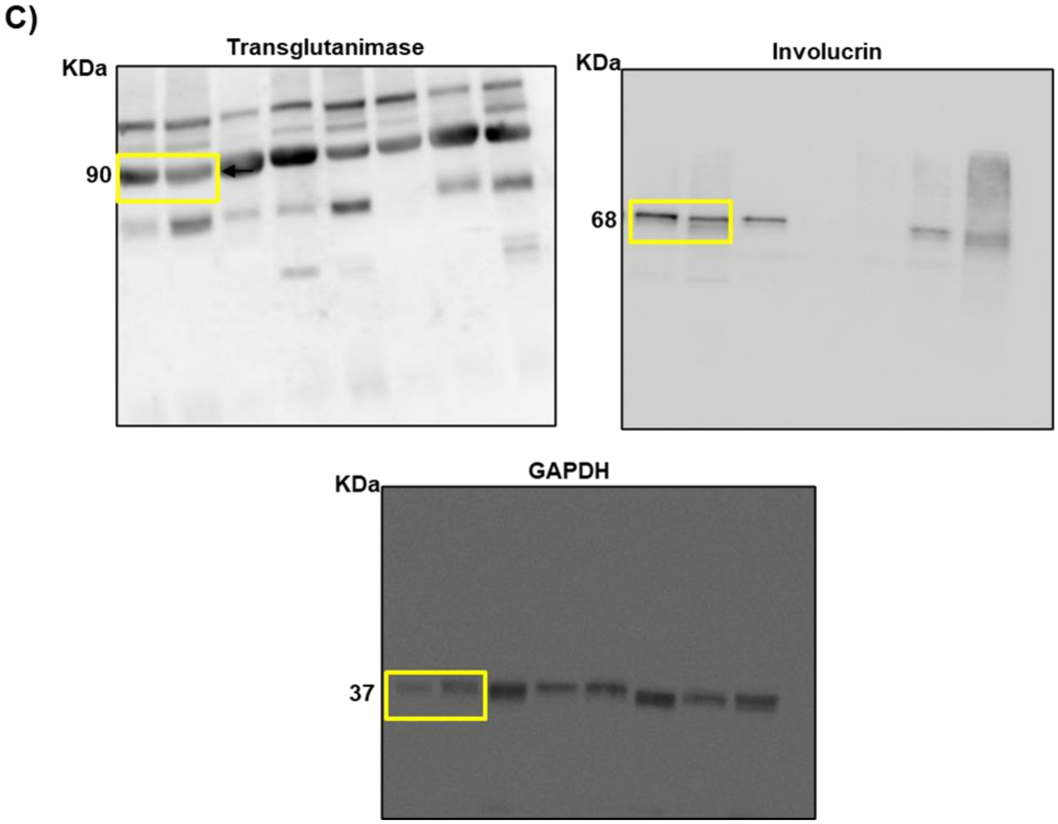

Figure S7C. Uncropped Western blot images
